# Decision support through risk cost estimation in 30-day hospital unplanned readmission

**DOI:** 10.1371/journal.pone.0271331

**Published:** 2022-07-15

**Authors:** Laura Arnal, Pedro Pons-Suñer, J. Ramón Navarro-Cerdán, Pablo Ruiz-Valls, Mª Jose Caballero Mateos, Bernardo Valdivieso Martínez, Juan-Carlos Perez-Cortes

**Affiliations:** 1 Instituto Tecnológico de Informática (ITI), Universitat Politècnica de València, València, Spain; 2 Health Research Institute of La Fe University Hospital, Fernando Abril Martorell, València, Spain; Universitat Politecnica de Catalunya, SPAIN

## Abstract

Unplanned hospital readmissions mean a significant burden for health systems. Accurately estimating the patient’s readmission risk could help to optimise the discharge decision-making process by smartly ordering patients based on a severity score, thus helping to improve the usage of clinical resources. A great number of heterogeneous factors can influence the readmission risk, which makes it highly difficult to be estimated by a human agent. However, this score could be achieved with the help of AI models, acting as aiding tools for decision support systems. In this paper, we propose a machine learning classification and risk stratification approach to assess the readmission problem and provide a decision support system based on estimated patient risk scores.

## Introduction

Unplanned hospital readmissions mean a great burden for health systems due to their repercussion on the quality of healthcare and their economic impact, as well as the direct consequences on the patients, who see their health deteriorated. At least in Europe, this threat is expected to worsen as the population increases in age, thus being more susceptible to develop comorbidities and chronic diseases. For these and other reasons, the number of hospital readmissions is currently among the most important indicators of quality of care [1].

It is believed that around 27% of readmissions that happen within 30 days of discharge from the initial admission date could be avoided or anticipated by more exhaustively monitoring the patients at risk [2–4]. However, making a human decision on which patients to profusely track and which not can be difficult because of the amount and heterogeneity of indicators, diseases, and clinical features with their corresponding interactions that come into play.

Having a patient’s score from 0 to 100 which served as a proxy for the risk of readmission would offer physicians and hospital resource managers interesting information that could be exploited in several ways. For instance, this score would allow for the creation of ordered lists sorted by readmission risk, which in turn would allow physicians for better scheduling of the monitoring visits, as well as providing a broader picture to dynamically adapt the allocation of clinical resources as the number of patients at risk or available resources change. On the other hand, it would also offer physicians additional information to decide the best moment for hospital discharge.

Foreseeing readmission episodes without this critical score can be very difficult for physicians. This is due to the high workload that brings taking a large number of decisions in a short time, and because of the great number of heterogeneous factors that must be considered during the decision-making process for every single patient.

The amount of heterogeneous factors that can affect the readmission risk for a patient makes it a typical task to tackle with multidimensional statistics and machine learning models, which would support physicians during the decision-making process. These techniques are not influenced by external factors, consistently offering the same prediction for the same input values. On the other hand, some techniques allow for a better understanding of the prediction process, disclosing the most relevant features, their specific effects on the results, or how they interact with each other. Besides, these can offer measures about prediction errors, which could help physicians know the risk associated with their decisions. Finally, a continued assessment of the estimated error can help to continuously improve the model by searching for new features or applying progressive adjustments to minimise this error.

Logistic regression models are the most common solution to estimate the hospital readmission risk. The two most often used models are LACE [5] and HOSPITAL [6, 7]. On the one hand, LACE accounts for: **L**ength of stay, **A**cuity of admission, **C**omorbidity score and **E**mergency dept. visits. On the other hand, HOSPITAL accounts for: **H**emoglobin before discharge (positive if < 12*g*/*dl*), discharge from an **O**ncology service, **S**odium level before discharge, **P**rocedure performed during hospitalisation, **I**ndex admission **T**ype, number of **A**dmissions in the previous 12 months, and **L**ength of stay.

Reviewing some metastudies in hospital readmission risk assessment [8–10] done at different points in time gives a broad idea of the evolution of the works in the field. Statistical regression models and traditional survival analysis methods, such as Cox regression models [11] and Kaplan-Meier estimators [12], which once dominated the field, are gradually being replaced by newer, more complex machine learning-based techniques beyond logistic regression.

It is difficult to select the best algorithm among the candidates, due to differences in design, approach and context of the different studies: patient cohorts, sample sizes, reproducibility across health systems or the focus on specific pathologies versus the general case. Most studies in the field (around 75%) estimate the overall model performance basing on the ROC-AUC [10]. A ROC-AUC value of 0.75 has not been surpassed by most models since 2011 [10], except for a few exceptional cases which we use as references for our model design. The work with the best-found results for general readmission causes, released in 2013, reports an AUC of 0.76 [13].

The remainder of this paper is structured as follows: in Section Dataset, the dataset used in this work is defined. Section Design and procedure describes how classification models are trained and evaluated using this data. Moreover, this section features a costs optimisation scenario which is proposed to prove the effectiveness of the trained models. Then, the results of the classification and cost optimisation cases are presented in Section Results and discussed in Section Discussion. In Section Conclusion the reached conclusions and future research in this area are disclosed.

## Materials and methods

### Ethics

This research was carried out with accordance with University and Polytechnic La Fe Hospital, which approved the study on 26th November 2019 under the name “Desarrollo de un modelo predictivo de ingresos y reingresos no programados a 30 días en el Departamento de Salud-Valencia La Fe” (Registration number: 2019–309-1). Patient privacy was maintained by using data previously pseudo anonymised with non-traceable codes and only authorised people obtained data from electronic health records. The Hospital’s Ethics Committee waived the requirement for written consent since data was pseudo anonymised and the study complies with national and European legal requirements regarding data protection.

### Dataset

This work was done in collaboration with La Fe Health Department and the Health Research Institute of La Fe University Hospital. Therefore, the dataset used during the experiments was build from a subset of patient information included in their Electronic Health Record (EHR) system. La Fe Health Department has deployed an EHR at different care levels, including over 20 million records, effectively organized reaching stage 6 in the eight-stage (0–7) EMRAM maturity model. Currently, the data lake layer includes structured and semi-structured information, coming from several information systems involving clinical activity, such as emergency care settings, outpatient, hospitalization, clinical reports, surgical unit, intensive care unit, hospital at home care. The data feeding the platform is composed by the aggregation of 22 datamarts and comprises 750 million rows, 84 tables, 4064 columns resulting in a total size of 640Gb. Data updates are scheduled on a daily, weekly, or monthly basis, depending on the datamart. This pseudo-anonymised and non-public dataset is protected by GDPR and spanish LOPDGDD laws.

The subset of data used contains information from 35034 episodes of 22370 patients. Data from five different categories was gathered and merged into one table:

- **Consumptions**: 10 features with information about the aggregated consumption of services and tests. These include previous visits to hospital, outpatient or urgency departments, among others.
- **Laboratory**: features with information from laboratory tests, such as results from urine or blood tests. Each feature contains the numerical result for a test. 462 features were selected according to clinical advice.
- **Treatments**: list of per patient active principles and time of treatment before and after the hospitalisation episode. Treatments were grouped by Anatomical Therapeutic Chemical (ATC) Classification System codes. 422 features were extracted from this data source.
- **Hospitalisation**: 53 features regarding hospitalisation episodes and their context, including admission diagnosis and procedure codes, length of stay and time of discharge, etc. This data also contained patient data, such as gender or age.
- **Comorbidities**: information on patient’s Charlson comorbidities [14] in a given episode and the time from diagnosis of comorbidity to the hospitalisation episode. 18 features (one feature for each comorbidity) were extracted.

As a result, a table of 35034 rows and 962 columns was obtained, where each row accounts for an admission episode. For each one of these episodes we know the date of admission and discharge. The observation period for this dataset ranges between 1/1/2015 and 30/12/2018. In order to train a classification model, the target variable takes unit value if readmission has taken place within 30 days following previous discharge, and zero otherwise. Following this rule, only 9.85% of the episodes in the dataset are positive class (readmissions).

### Design and procedure

In this section, the data processing and model selection, training and evaluation processes are described.

Prior to model training, the dataset described in Section Dataset is processed. Laboratory features were reduced to 46 according to clinical advice. Treatments that occur within a time window of 90 days prior to the hospital admission are considered, as well as those prescribed during the hospital stay and discharge. Then, these were grouped in higher levels of ATC, resulting in a total of 36 treatment features. Two more features are computed by counting the number of prescription drugs before and after the hospitalisation episode. Lastly, one hot encoding was applied to binnarize categorical features with three or more categories, such as diagnostic and procedure codes.

Missing values are common in EHR, so feature values must be imputed or discarded if missing value count is high. For this task, first, columns with a missing value percentage greater than 30% are removed. In the remaining laboratory columns, imputation quality is assessed by replacing 30% of the values with missing ones and then imputed, measuring the R2-score between the true and guessed value arrays. Then, columns which have less than 30% but more than 15% missing values and an R2-score lesser than 0.5 are also removed. The remaining missing values are imputed depending on the feature’s source. Laboratory features are imputed using the iterative method found in *scikit-learn* [15] implementing the Bayesian Ridge algorithm as the regression model [16]. In contrast, missing values in consumption, comorbidities or treatment features are imputed with zeros, meaning the absence of that feature.

Pearson correlation coefficient is then calculated for each possible pair of features, and one of each pair that surpasses a value of 0.9 is filtered out. This action is performed recursively until no pairs have a correlation coefficient above the said threshold. Moreover, invariant features, that is to say, those where more than 99% of the values are equal to the mode value, are removed. Lastly, features with a variance inflation factor (VIF) greater than 10 are also filtered out.

After performing all the aforementioned preprocessing steps, as well as removing duplicates, the dataset contains 35034 rows and 200 features. Tables 1–3 show summary statistics for each numerical, categorical and binary feature. Note that categorical features are transformed using one-hot encoding, so the final number of features is higher than those shown in these tables. S1 Appendix provides a short description of these features.

**Table 1:** Numerical features: Summary statistics.

Feature name	Mean	STD	Q1–Q3
lengthofstay	7.035	7.262	3.0–8.0
age	68.136	18.87	57.0–83.0
count_external	0.156	0.524	0.0–0.0
count_imaging	0.646	1.809	0.0–1.0
count_cex	26.588	47.467	3.0–36.0
count_hosp	2.008	3.038	0.0–3.0
count_urg	7.155	8.387	2.0–9.0
count_ecgs	0.929	1.167	0.0–1.0
count_octests	0.33	1.077	0.0–0.0
count_surgery	0.204	0.615	0.0–0.0
pro_bnp_pg/ml	3810.276	6530.72	396.55–3991.25
hemoglobin_g/dl	12.097	2.115	10.5–13.6
leukocytes 10^3^/l	9.057	6.369	6.34–10.7
pco2_arterial_mm_hg	44.498	12.047	36.3–50.4
neutrophils_%_%	67.465	13.251	59.2–76.8
hematocrit_%	37.233	6.011	32.9–41.6
ph_venous_nounit	7.384	0.06	7.35–7.42
leukocytes_/l	106.383	182.531	0.0–100.0
basophils_%_%	0.438	0.336	0.2–0.6
lymphocytes 10^3^/l	1.752	3.868	1.07–2.09
chlorine_meq/l	101.584	4.731	99.0–104.6
CRP_mg/l	46.341	63.618	5.61–60.062
pco2_venous_mm_hg	43.15	8.542	37.5–47.9
creatinine_mg/dl	1.088	0.946	0.69–1.15
po2_venous_mm_hg	51.583	31.228	31.8–60.05
MCV_fl	90.195	6.708	86.5–94.1
eosinophils 10^3^/l	0.172	0.231	0.04–0.24
alt/gpt_u/l	31.402	71.666	11.9–29.3
eosinophils_	2.192	2.421	0.5–3.1
albumin_g/dl	3.542	0.559	3.17–3.94
basophils 10^3^/l	0.039	0.332	0.02–0.05
potassium_meq/l	4.188	0.535	3.86–4.5
monocytes 10^3^/l	0.774	0.544	0.53–0.93
ckd_epi_ml/min	74.927	29.52	54.0–96.0
sodium_meq/l	140.027	3.879	138.0–142.0
rdw_cv_%	14.657	2.313	13.2–15.5
ph_arterial_nounit	7.409	0.062	7.38–7.448
po2_arterial_mm_hg	71.104	27.473	56.0–78.1
total_comorbidities	1.796	1.792	0.0–3.0

For each feature, mean, standard deviation and the range between percentile 25% and percentile 75% are shown.

**Table 2:** Categorical features: Summary statistics.

Feature name	#Categories	Most common (%)	Least common (%)
ccsr_dx	21	40.62%	2.064%
ccsr_px	27	85.325%	0.0%
charlson_code	17	8.216%	0.891%
prev_route_admin	12	4.392%	7.397%
post_route_admin	7	12.289%	1.166%
cod_reason_admission	19	84.824%	0.004%
cod_service_admission	64	12.468%	0.004%
cod_nursingunit_admission	33	10.669%	0.004%
cod_realservice	68	12.103%	0.004%
cod_reason_discharge	4	87.128%	0.011%
cod_service_discharge	67	12.103%	0.004%
cod_nursingunit_discharge	32	11.363%	0.004%
cod_destination_discharge	12	45.889%	0.018%
cod_service_destination	111	7.54%	0.004%
month_admission	12	9.982%	7.5%

For each feature, the number of categories, as well as the frequency of the most and least common values, are shown.

**Table 3:** Binary features: Summary statistics.

Feature name	Freq. 0 (%)	Freq. 1 (%)
sex	46.64%	53.36%
readmission30d	9.65%	90.35%
prev_n02be	16.539%	83.461%
prev_n06ab	5.612%	94.388%
prev_h02ab	6.778%	93.222%
prev_b01ab	4.392%	95.608%
prev_m04aa	4.124%	95.876%
prev_r03al	3.28%	96.72%
prev_j01dc	3.816%	96.184%
prev_a11cc	5.462%	94.538%
prev_other_atc0_a	21.743%	78.257%
prev_other_atc0_b	9.421%	90.579%
prev_other_atc0_c	20.566%	79.434%
prev_other_atc0_other	8.963%	91.037%
post_a02bc	5.261%	94.739%
post_h02ab	7.858%	92.142%
post_other_atc0_r	5.0%	95.0%

For each feature, the frequency of the null and unit values is shown.

The class imbalance is redressed using random undersampling of the majority class and ADASYN [17] oversampling of the minority class in different instances, whose results will be later compared. Only training samples selected in each fold are resampled in the balancing process, in order to avoid the introduction of biases in the test data. No other performance-based feature selection nor feature extraction methods are used in this study.

Three ensemble methods are chosen for the binary classification task:

- Random Forest (RF) [18] is an ensemble supervised learning method which incorporates many weak decision tree models. During the training phase, each of these individual trees sees a random set of features, ensuring some level of individuality. To make a prediction, each of these trees makes its guess and then a voting phase takes place to select the ensemble’s final decision.
- Gradient Boosting (GB) [19] is also a decisiong tree ensemble technique, not bagged horizontally but in a stage-wise manner known as *boosting*. Arranged like a chain, each tree’s training parameters depend on the result of the previous one, optimising the performance in the later tree.
- Extreme Gradient Boosting (XGBoost) [20] is an enhanced, highly efficient and computationally effective implementation of the GB algorithm. Moreover, XGBoost is regularized in a way which usually lends to better results and prevents overfitting.
- Additionally, decision tree models are trained in order to compare the performance of the previous with that of a simpler one.

In this study, samples are distributed between train and test sets using nested 10-fold cross-validation. After the model is trained in each split, performance metrics are calculated on the corresponding test set. When this procedure is over, these metrics will be averaged to get an overall model performance.

After classification, a probability calibration pipeline is also implemented in order to assert that class probabilities yielded by models match a better estimation of the real-world probabilities. This technique is not intended to enhance the overall model performance, but to increase its results understandability and its aiding power on decision-making (i.e. knowing at which level of risk the discrimination threshold would be set). The tested calibration methods are Isotonic and Beta Calibration [21], an enhanced version of the classical Platt Scaling for binary classifiers. Probability calibration’s goodness of fit is assessed *via* Brier Score and Log-Loss [22]. Both metrics accounting for errors, the best calibration would be the one obtaining the lesser score. Although similar in that regard, Log-Loss will give greater weight to errors in the lesser probabilities. These will be later reviewed in the Results section and a definitive calibrator will be chosen.

Once the probabilities are calibrated, all test cases are stratified based upon the percentiles to which their probabilities belong. Then, these percentiles, which range from 0 to 100, can be divided into different readmission risk tiers. Percentile and probability ranges give different information that can be used for different ends. Taking the very same readmission problem as an example, based on the data available in this study, the *a priori* probability of readmitting before 30 days after discharge is around 9.8%. Thus, while a predicted probability of 20% may intuitively seem to fall on the low end, it would actually pertain to the highest risk percentiles as it is much higher than the base risk.

### Cost analysis

**Fig 1:**
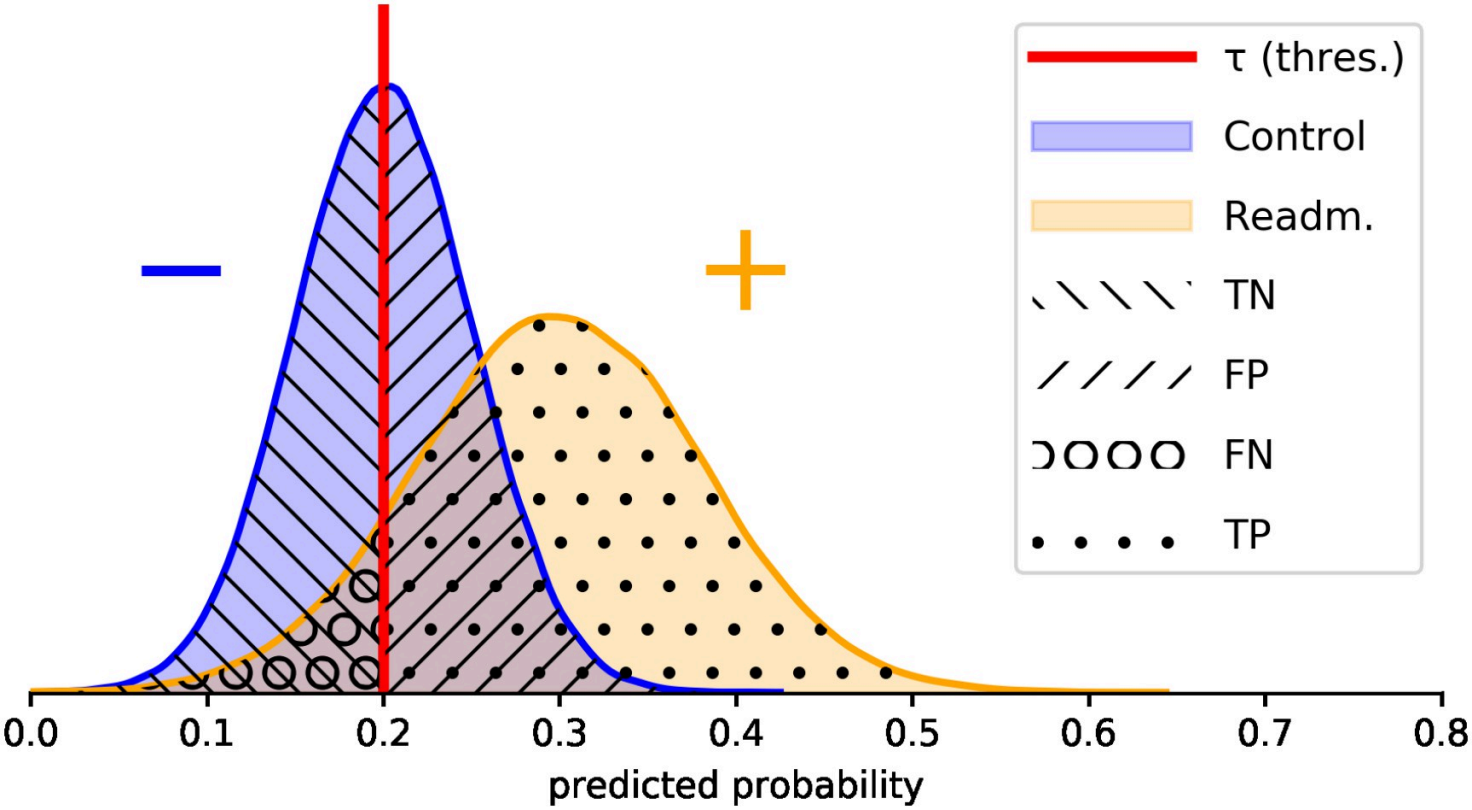
Probability distribution of the output of a model for controls and readmission cases. False Positives (FP), true negatives (TN), false negatives (FN) and true positives (TP) are depicted over their respective class distributions. Threshold *τ* establishes the risk score from which patients are classified between control and readmission. Example case with synthetic data.

Finally, a costs-optimization scenario will be simulated in order to better understand the effectiveness of implementing readmission prevention plans based on the information given by a predictive model, under a cost analysis point of view. Fig 1 shows two fictional probability distributions: the left (blue) one corresponds to negative cases, e.g. patients than do not require readmission within 30 days after discharge. Correspondingly, the right (yellow) one belongs to positive cases, e.g. patients that do require readmission.

As it is shown in Fig 1, in the case that a model is not perfect, the probability distribution of both classes would appear overlapped. A chosen threshold *τ* establishes the risk score from which patients that lay at the left will be discharged from the hospital, and patients that lay at the right will be exhaustively monitored or will stay at the hospital. Since the distributions overlap, several errors will be made in both directions (FN, FP).

An easily understandable decision support system is proposed by means of a unidimensional objective function related to a cost that varies as a function of the established threshold. We define two different cost functions (*F*_*h*_, *F*_*d*_) whose addition will explain the total cost for the public healthcare system (*F*_*t*_ = *F*_*h*_ + *F*_*d*_). The function *F*_*h*_ corresponds to the cost of patients that stay at the hospital (*h*) or are exhaustively monitored (readmission prevention plans), while *F*_*d*_ corresponds to the patients that are discharged (*d*), some of whom then require readmission. Let *c*_1_ be the cost for an individual that enters the readmission prevention plan, *c*_2_ the cost per patient that is readmitted after a discharge and finally *c*_3_ the cost for discharged patients that do not require readmission. *c*_3_, which acts as a baseline cost, reflects the use of non-hospitalisation resources, such as medication. For the sake of simplicity, *c*_2_ and *c*_3_ will be defined as multiples of *c*_1_: *c*_2_ = *n*_2_ ⋅ *c*_1_, *c*_3_ = *n*_3_ ⋅ *c*_1_, considering *n*_2_ > 1, *n*_3_ < 1. Note that the *n*_2_ limit implies that the cost of managing a patient’s readmission is higher than performing a prevention plan, as stated by the asked physicians.

Under these assumptions, plus assuming all readmissions as potentially avoidable, the costs for a given threshold (*τ*) can be computed as in Eqs 1 and 2.

$$\begin{array}{c} \begin{array}{cc} F_{h} & =c_{1}(FP+TP)\\ F_{d} & =c_{2}(FN)+c_{3}(TN)\\ F_{t} & =F_{h}+F_{d} \end{array} \end{array}$$ (1)

$$\begin{array}{c} \begin{array}{cc} F_{t} & =c_{1}(FP+TP)+c_{2}(FN)+c_{3}(TN)\\ & =c_{1}(FP+TP)+n_{2}c_{1}(FN)+n_{3}c_{1}(TN)\\ & =c_{1}(FP+TP+n_{2}FN+n_{3}TN) \end{array} \end{array}$$ (2)

For each cost factor *n*_2_, gradually changing the decision threshold will yield a costs curve whose bottom point will correspond to the optimum threshold. In a sense, this is equal to optimising cost matrices with different cost ratios.

### Software

In this study we used Python packages frequently used in machine learning. We used scikit-learn’s [15] implementation of DT, RF and GB models, and xgboost’s [20, 23] implementation of XGBoost. For missing data imputation, we used scikit-learn’s IterativeImputer and SimpleImputer. We also used scikit-learn’s permutation importance [18] procedure to evaluate feature importance. For calibration, we used the betacal library [21] and scikit-learn for IsotonicCalibration. For oversampling and subsampling, we used the imbalanced-learn library [24]. For other processing procedures, pandas [25, 26] and numpy [27] were used. Plots and figures were built using matplotlib [28] and seaborn [29] packages.

## Results

### Binary classification

**Fig 2:**
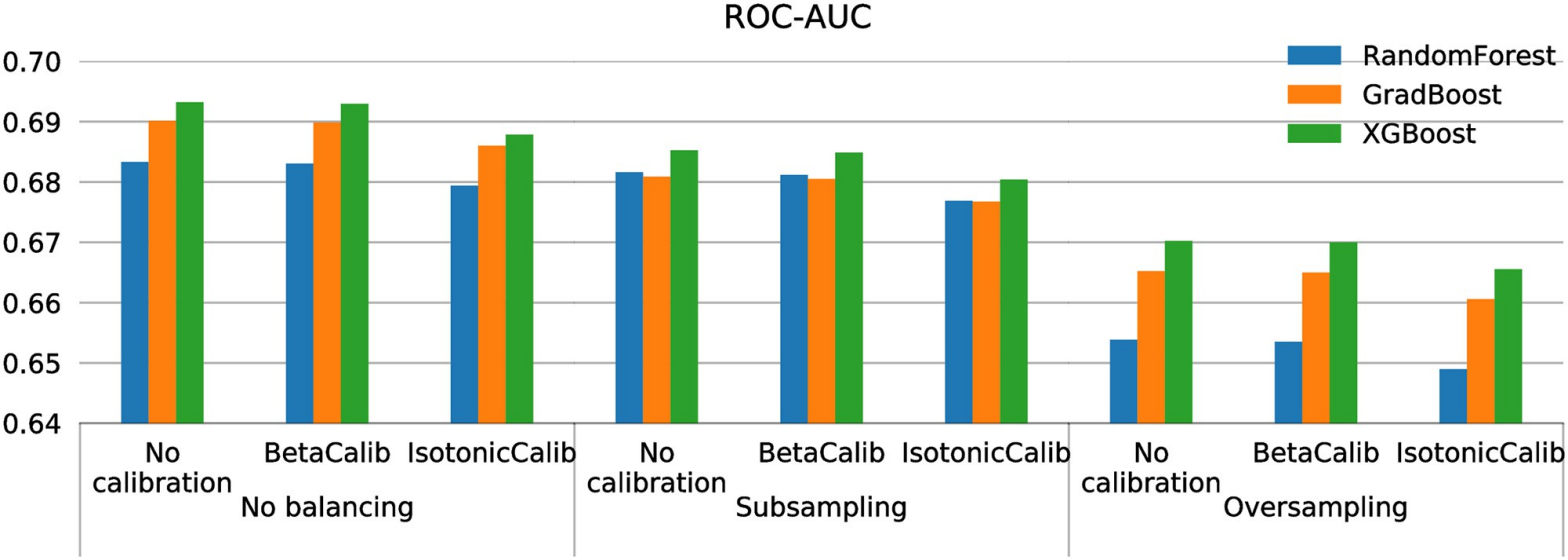
ROC-AUC with different models, balancing and calibration methods.

All results in this section are computed performing nested 10-fold cross-validation on the entire dataset and averaging the resulting metrics computed on each test set. The best ROC-AUC results are obtained with XGBoost models and no balancing method (Fig 2). Moreover, the best calibration is achieved performing Beta Calibration, as it shows both the lowest BrierScore and LogLoss metrics in Table 4. While the ROC-AUC varies slightly with different calibration options, improving the model performance is not a target of this task. The calibration step aims to scale the prediction results to a more understandable, truthful class probability range, but does not enhance class separability.

**Table 4:** Results with different balancing methods (no balancing, random majority subsampling and ADASYN oversampling) and calibration methods (no calibration, Beta Calibration and Isotonic Calibration).

Balancing	Calibration	BrierScore	LogLoss	ROC-AUC
No balancing	No calibration	0.08517	0.30110	0.69321
	BetaCalib	0.08513	0.30087	0.69296
	IsotonicCalib	0.08518	0.30290	0.68784
Subsampling	No calibration	0.22497	0.63911	0.68525
	BetaCalib	0.08544	0.30239	0.68489
	IsotonicCalib	0.08556	0.30368	0.68040
Oversampling	No calibration	0.08774	0.31154	0.67023
	BetaCalib	0.08598	0.30549	0.66999
	IsotonicCalib	0.08602	0.30650	0.66555

All results in this table are obtained with XGBoost, 10-fold cross-validation.

**Fig 3:**
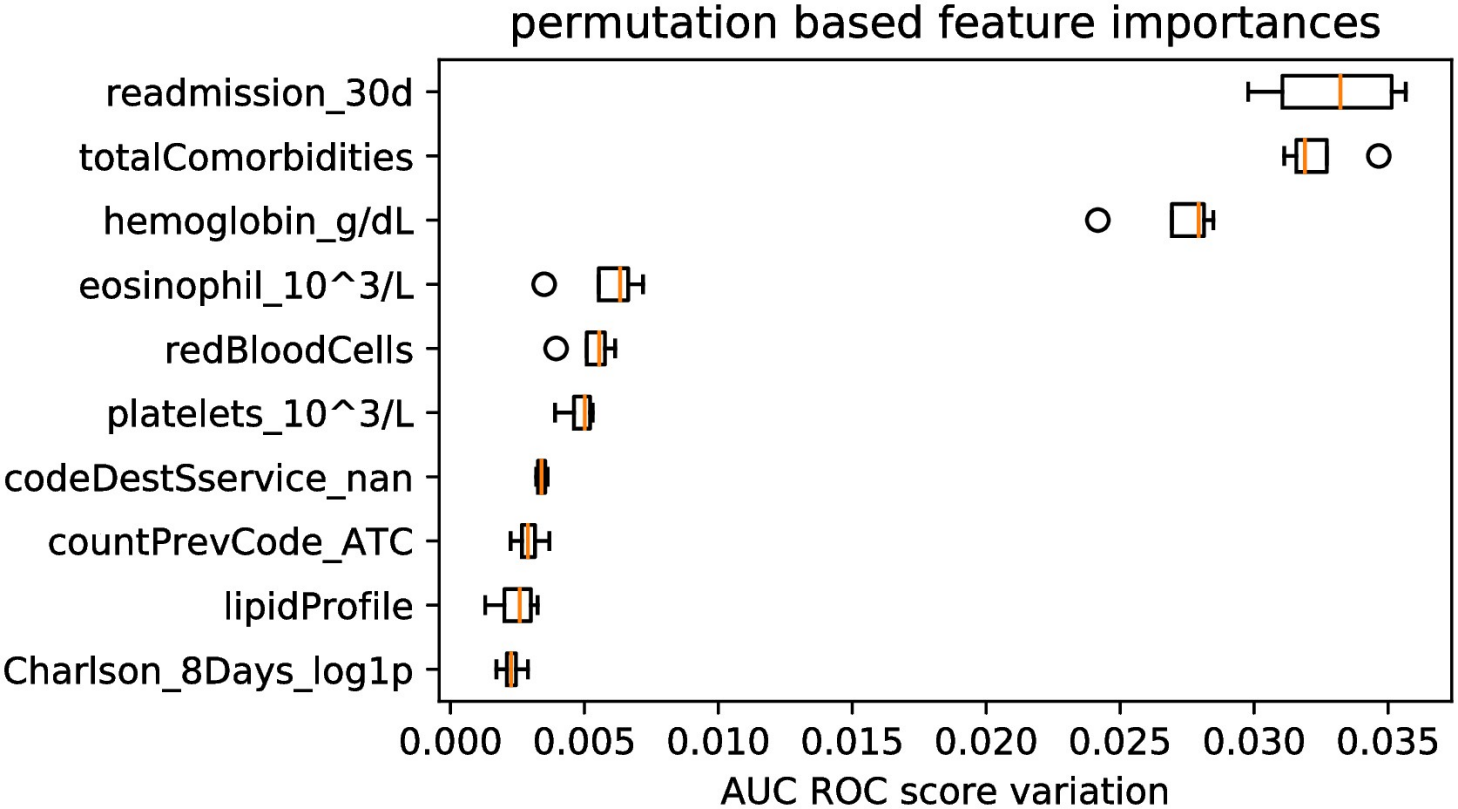
Top ten features sorted by permutation importance in the XGBoost model.

The top ten most important features in this model are shown in Fig 3, where three features stand out over the others:

- *readmission_30d*: whether or not the past admission was a readmission. Whilst ∼ 20% of cases where this feature takes positive value end up in readmission, only ∼ 9% of cases where the value is zero do, which suggests successive readmission are relatively common.
- *totalComorbidities*: the total number of comorbidities a patient has at the time of admission.
- *hemoglobin_g/dL*: laboratory measurement of the amount of haemoglobin in blood. Anaemia, described as a low presence of haemoglobin in blood, is often associated with some of the most common causes of readmission [30, 31].

**Fig 4:**
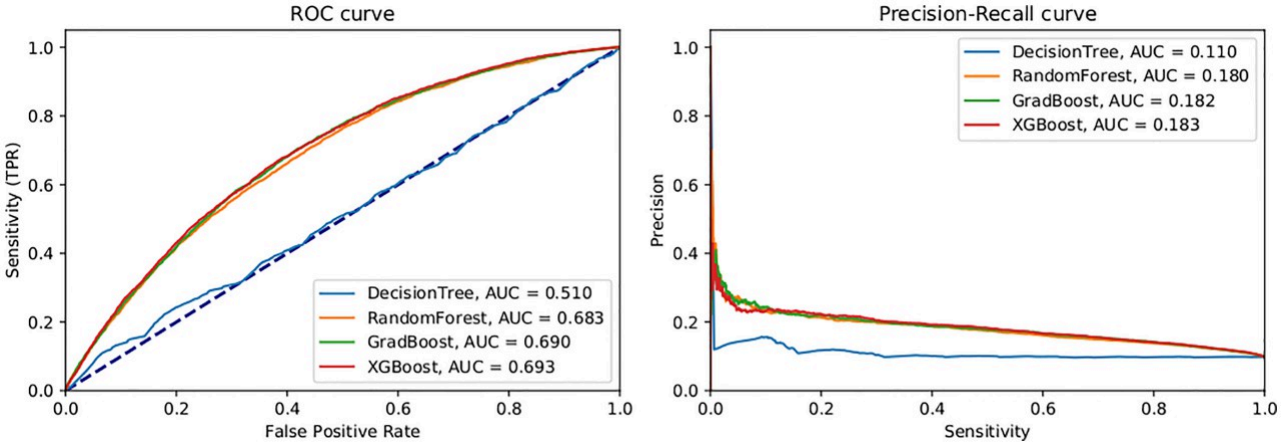
Comparison of model performance curves. For each one of the machine learning models, the left graph shows the receiver operating characteristic (ROC) curve, while the right shows the precision (PPV) versus recall (TPR) curve. No balancing was applied, since it led to the best results.

Fig 4 shows the ROC and Precision-Recall curves for all trained models. Since both curves (especially the latter) show low profiles for any decision threshold *τ*, there is no obvious cut-off point to choose. Table 5 shows true positives and negatives counts when the decision threshold is set at different probability percentiles. The Positive Predictive Value is also shown for each one of the thresholds. Note that the matching calibrated probabilities to each threshold are low from the bottom to the top percentiles, with percentile 90% still falling under a 20% probability.

**Table 5:** Metrics at different readmission probability thresholds *τ*.

Percentile	Threshold *τ*	TP	Sensitivity	TN	Specificity	PPV
10%	0.03	3383	0.981	3438	0.109	0.107
20%	0.04	3263	0.946	6821	0.216	0.116
30%	0.06	3095	0.897	10156	0.322	0.126
40%	0.07	2884	0.836	13449	0.426	0.137
50%	0.08	2590	0.751	16658	0.527	0.148
60%	0.10	2264	0.656	19835	0.628	0.162
70%	0.12	1858	0.539	22933	0.726	0.177
80%	0.15	1353	0.392	25931	0.821	0.193
90%	0.19	764	0.222	28845	0.913	0.218

Thresholds matching each calibrated probability decile are shown with their corresponding true positive (TP) and true negative (TN) counts, sensitivity (TPR), specificity (TNR), as well as their Positive Predictive Value (PPV).

If the problem is approached as a risk stratification task instead, different probability cut-off points could be used based on the specific needs of a given hospital at a given point in time. Table 6 shows how different probability percentile ranges contain readmission and control cases in very different proportions. While readmissions become increasingly predominant as we move towards higher percentiles, control cases still constitute the vast majority even in the upper tiers, which is indicative of an overall low precision score.

**Table 6:** Percentage of patients whose true target is positive, i.e. readmitted before 30 days after discharge, at different percentile ranges of predicted risk.

Percentiles	% Readmission
0–10%	1.88
10–20%	3.43
20–30%	4.80
30–40%	6.02
40–50%	8.39
50–60%	9.31
60–70%	11.59
70–80%	14.42
80–90%	16.81
90–100%	21.80

All records in the dataset are used for this table, merging test predictions of a 10-fold cross-validated experiment to sort patients based on their predicted risk. For reference, the *a priori* < 30*d* readmission rate in the complete dataset is around 9.8%.

### Cost analysis

**Fig 5:**
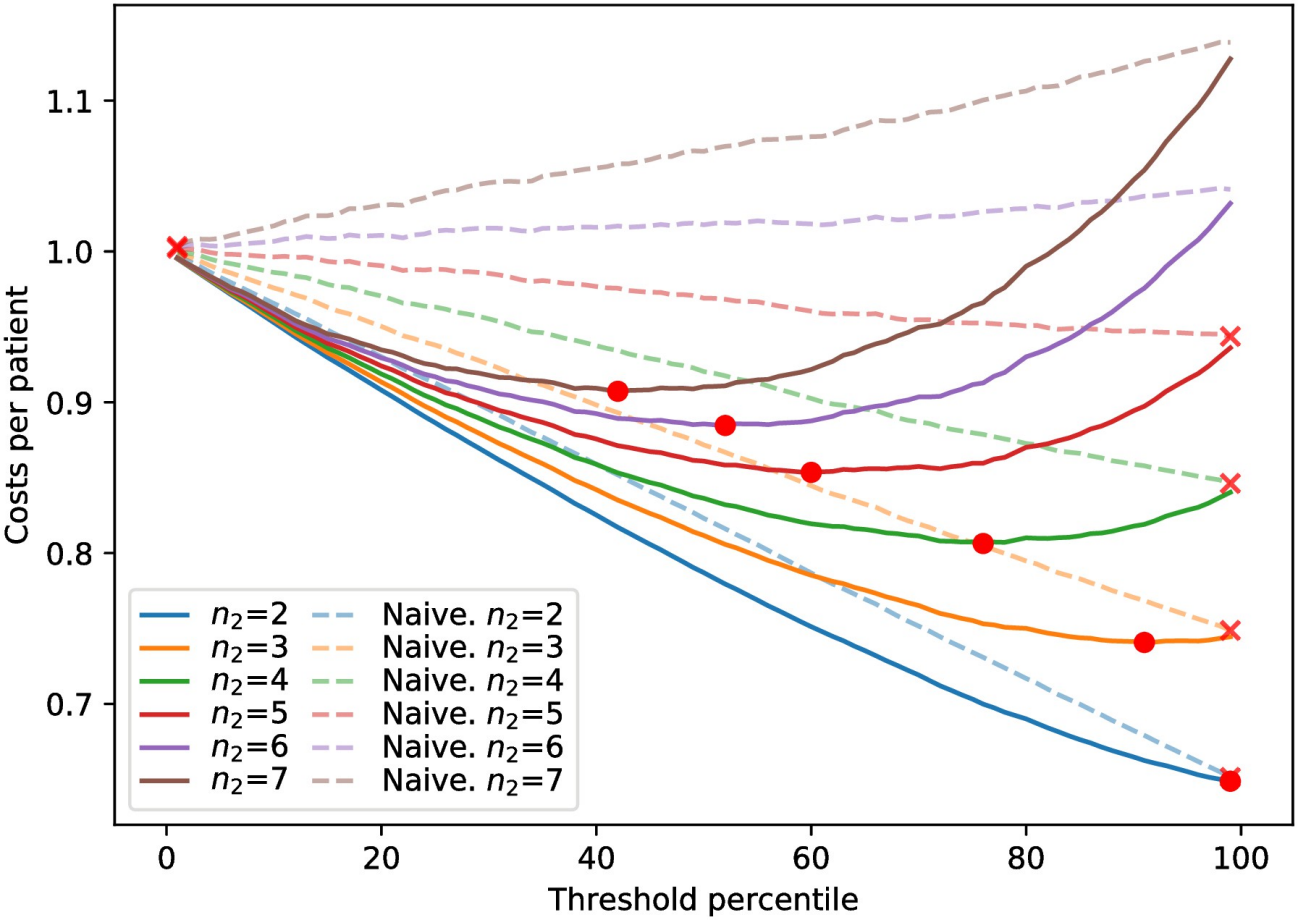
Costs per patient at different decision thresholds and readmission costs, modelled by *n*_2_. Points and crosses mark optimum cost in our model and in the naive model, respectively.

Fig 5 shows costs per patient when setting the decision threshold at different risk percentiles. Each pair of lines accounts for a different value of *n*_2_, where the solid line represents our own model and the dashed line corresponds to a naive model (*ROCAUC* = 0.5). The value of *c*_3_ is set at half of *c*_1_ at all times (*c*_3_ = *n*_3_ ⋅ *c*_1_, *n*_3_ = 0.5). Recall that cost factors *n*_2_ and *n*_3_ in Eq 2 are parameters to indicate that costs are multiples to one another. These costs should be set to the real costs that exist at each hospital. This Figure is further discussed at Section Discussion.

## Discussion

The best classification results are obtained with an XGBoost model, with a better match between predicted and real class probabilities after performing Beta Calibration (*ROCAUC*: 0.693, *BrierScore*: 0.0851, *LogLoss*: 0.3009). Besides, classification metrics have great room for improvement in terms of both recall and precision. Furthermore, the best results are obtained when no class balancing is performed, but it is worth noting that the subsampling approach yielded notably better results than the oversampling one. This might suggest there is no need for more general data, but it will help to have more minority class (readmissions) data.

Regarding probability calibration, the low values matching each percentile observed in Table 5 show that the probability spectrum agglomerates within a low range, probably due to a natural class imbalance that reflects in the test dataset. Redressing this imbalance to spread the readmission probability across the 0 to 1 axis could introduce a bias altering results in real setups.

Analysing Table 6, it can be seen that even in the top risk percentile ranges, the proportion of control cases is still considerably large compared to that of readmission cases. However, it is worth remarking that the proportion of readmissions in the top level is more than ten times larger than in the bottom level, evidencing an effective class separation. Since the *a priori* <30 *d* readmission probability is around 9.8%, cases predicted in the lower decile range are around 5 times less likely to be positive class than the general case, while those in the upper decile range are around 2 times more likely.

In Section Introduction, the need for a score, preferably between 0 and 100, was stated. Although the class probabilities do not range between these limits, risk percentiles do. Therefore, taking into account previous patient data, the probability percentile (0–100%) assigned to a patient could constitute a score with a clear real-life meaning in which patients can be ranked along.

Lastly, the cost analysis results previously shown in Fig 5 are analysed. It can be seen that, as the difference between *c*_1_ (readmission prevention acting) and *c*_2_ (unplanned readmission) grows, the optimal minimum is set at a lower risk. When comparing the performance of our model (a solid line for each *n*_2_) with the naive model (dashed line), it appears that the costs when making decisions based on our model’s predictions are always lower, being only equal at extreme points where randomness does not play any effect (selecting all or none patients). Moreover, the random model’s optima only fall at 100% (no prevention planning whatsoever) or at 0% (all patients included in the prevention plan) with the highest costs of readmission. It is worth mentioning that the cost reduction achieved using our model instead of the naive model greatly increases as the readmission costs grow.

## Conclusion

Thoroughly gathering patient data and hospital indicators is critical to reducing readmission rates. A model like the ones proposed here could seize this data to aid in foreseeing avoidable episodes, helping in managing and optimising hospital resource utilisation. Despite the limited predictive power shown by classification models, probably due to the lack of readmission data at the moment of writing, accurately ranking patients by estimated risk could allow improving resource allocation for those in the top levels of risk. As shown, cost-effectiveness at different risk decision thresholds could variate greatly depending on cost ratios. However, despite this study still lacks clinical validation, the obtained results suggest that the predictions made by the currently proposed models would always lead to equal or lower costs per patient.

### Future work

The performance of the prediction models could be improved by adding the dataset a collection of socio-demographic variables available for most of the patients. We expect that these variables would explain some of the high variance that perhaps our current data does not grasp. It should be composed of information as the presence of home companions or sitters, socioeconomic status, location, etc. If some of these variables were inaccessible, median information of a population based on the known approximate location, neighbourhood or postal code could be used.

A machine learning-based survival analysis approach could prove to perform as well as the classification models presented in this paper, if not better, while providing great flexibility in its possible use cases. In this approach, features would act as regress covariates against time until readmission, and assess the risk of each patient being readmitted within any specified number of days after discharge. Unlike traditional regression methods, survival models are tailored to take into account data censoring (e.g. patients not yet readmitted as of the end of the study period). In the classification approach, two patients who were readmitted at 29 and 31 days after discharge, while probably having similar risks, are assigned opposite classes. This can difficult the class separation during training. However, a survival analysis model is not as heavily affected by this minor difference in days since discharge, which could be beneficial in the training steps.

A suited cost analysis like the one described in Section Cost analysis could be as well developed for a survival analysis model, based on the cumulative hazard function for each patient. One potential improvement achievable by taking this alternate approach is the possibility of finding the optimal time duration from discharge to next admission that defines a readmission episode. If the 30-days restriction is lifted, an alternate period that optimises the accuracy of predictions or minimises costs based on these predictions could be set up. If this new period is in turn used for the classification task, it could potentially reduce the imbalance issue if it happened to be set above the 30 days limit. Note that more episodes would be considered hospital readmissions in this case.

Future research also includes implementing extended feature selection and extraction pipelines prior to the training step. Moreover, if more data could be acquired, a pre-clustering step could be included in order to group patients with a similar diagnosis or procedure codes together, assuming the features of individuals with the same code are in general closer, or interact differently from those with other codes. With the current dataset, many of the resulting groups end up heavily unpopulated and have high variance presumably due to lack of data. This high variance also hinders the performance of a hierarchical clustering step to agglomerate similar codes together.

## Supporting information

S1 Appendix List and description of dataset features. Brief description of the meaning of each feature used in the models.
(PDF)

S2 Appendix Additional information on the used methods. Detail of the main steps that compose the procedures mentioned in the methodology section.
(PDF)
