# Supplementary material for: Decision support through risk cost estimation in 30-day hospital unplanned readmission
Source: PLoS One. 2022 Jul 15;17(7):e0271331. doi: 10.1371/journal.pone.0271331 (PMC9286269; doi:10.1371/journal.pone.0271331)
Supplement: S1 Appendix — Brief description of the meaning of each feature used in the models. (PDF) [file pone.0271331.s001.pdf]

Decision support through risk cost estimation in 30-day hospital unplanned readmission.

S1 Appendix: List and description of dataset features

Laura Arnal[1◑*], Pedro Pons-Suñer[1◑], J.Ramón Navarro-Cerdán[1◑], Pablo Ruiz-Valls[1◑], Mª Jose Caballero Mateos[2◑], Bernardo Valdivieso Martínez[2◑], Juan-Carlos Perez-Cortes[1◑],

1 Instituto Tecnológico de Informática (ITI), Universitat Politècnica de València, Camino de Vera, s/n, 46022 València, Spain
2 Health Research Institute of La Fe University Hospital, Fernando Abril Martorell, Torre A, s/n, 46026 València, Spain

◑These authors contributed equally to this work.
* larnal@iti.es

Dataset description

In this section, features used in this study are paired with their respective meaningful descriptions. For convenience and consistency, they are shown in three different tables as in section "Design and procedure" of the manuscript:

- Numerical features (Table S1).
- Categorical features (Table S2).
- Binary features (Table S3).

Table S1. Numerical features: feature description.

Feature name	Description
lengthofstay	Length of stay (days)
age	Age of the patient at admission
count_external	Total number of pathology tests during the episode
count_imaging	Total number of imaging tests during the episode
count_cex	Total external consultings before the episode (90 days)
count_hosp	Total admissions before the current episode
count_urg	Total urgency consultations before the current episode
count_ecgs	Total electrocardiograms during the episode
count_other_tests	Other tests done before the episode
count_surgery	Total surgery procedures during the episode
pro_bnp_pg/ml	Lab result: B-type natriuretic peptide (PRO-BNP) (pg/mL)
hemoglobin_g/dl	Lab result: Hemoglobin_g/dL
leukocytes 10^3/l	Lab result: Leukocytes (10^3/L)
pco2_arterial_mm_hg	Lab result: Arterial PCO2 (mmHg)
neutrophils_%_%	Lab result: Neutrophils (%_%)
hematocrit_%	Lab result: Hematocrit (%)
ph_venous_nounit	Lab result: Venous PH (No unit)
leukocytes_/l	Lab result: Leukocytes (/L)
basophils_%_%	Lab result: Basophils (%_%)
lymphocytes 10^3/l	Lab result: Lymphocytes (10^3/L)
chlorine_meq/l	Lab result: Chlorine (mEq/L)
CRP_mg/l	Lab result: C-reactive protein (mg/L)
pco2_venous_mm_hg	Lab result: Venous PCO2 (mmHg)
creatinine_mg/dl	Lab result: Creatinine (mg/dL)
po2_venous_mm_hg	Lab result: Venous PO2(mmHg)
MCV_fl	Lab result: Mean corpuscular volume (MCV) (fL)
eosinophils 10^3/l	Lab result: Eosinophils (10^3/L)
alt/gpt_u/l	Lab result: ALT/GPT (U/L)
eosinophils_	Lab result: Eosinophils (%_%)
albumin_g/dl	Lab result: Albumin (g/dL)
basophils 10^3/l	Lab result: Basophils (10^3/L)
potassium_meq/l	Lab result: Potassium (mEq/L)
monocytes 10^3/l	Lab result: Monocytes (10^3/L)
ckd_epi_ml/min	Lab result: Glomerular filtration (CKD-EPI) (mL/min)
sodium_meq/l	Lab result: Sodium (mEq/L)
rdw_cv_%	Lab result: RDW-CV (%)
ph_arterial_nounit	Lab result: Arterial PH (No unit)
po2_arterial_mm_hg	Lab result: Arterial P02 (mmHg)
total_comorbidities	Number of total comorbilities of a patient.

List of numerical features paired with a meaningful description.

Table S2. Categorical features: feature description.

Feature name	Description
cod_reason_admission	Reason for admission
cod_service_admission	Code of service of admission
cod_nursingunit_admission	Code of nursing unit at admission
cod_realservice	Real service code
cod_reason_discharge	Reason for discharge
cod_service_discharge	Service at the moment of discharge
cod_nursingunit_discharge	Code of nursing unit at discharge
cod_destination_discharge	Destination after discharge
cod_service_destination	Service of destination
ccsr_dx	CCSR code for ICD-10-CM Diagnoses
ccsr_px	CCSR code for ICD-10-CM Procedures
charlson_code	Charlson comorbidity code
month_admission	Month of admission

List of categorical features paired with a meaningful description.

Table S3. Binary features: feature description.

Feature name	Description
sex	sex of the patient
readmission30d	The current episode is a < 30 day readmission itself
prev_n02be	Anilidas. Prescribed during or before the admission.
prev_n06ab	Selective serotonin reuptake inhibitors (SSRIs). Prescribed during or before the admission.
prev_h02ab	Glucocorticoids Prescribed during or before the admission.
prev_b01ab	Heparin group. Prescribed during or before the admission.
prev_m04aa	Preparations inhibiting uric acid production Prescribed during or before the admission.
prev_r03al	Adrenergics in combination with anticholinergics incl. triple combinations with corticosteroids. Prescribed during or before the admission.
prev_j01dc	Second-generation cephalosporins. Prescribed during or before the admission.
prev_a11cc	Vitamin D and analogues. Prescribed during or before the admission.
prev_other_atc0_a	Products acting on the alimentary tract and metabolism. Prescribed during or before the admission.
prev_other_atc0_b	Products acting on blood and blood forming organs. Prescribed during or before the admission.
prev_other_atc0_c	Products acting on the cardiovascular system. Prescribed during or before the admission.
prev_other_atc0_other	Other product with unknown ATC0 or not listed. Prescribed during or before the admission.
post_a02bc	Proton pump inhibitors. Prescribed at discharge.
post_h02ab	Glucocorticoids Prescribed at discharge.
post_other_atc0_r	Products acting on the respiratory system. Prescribed at discharge.

List of binary features paired with a meaningful description.
