# Supplementary material for: Decision support through risk cost estimation in 30-day hospital unplanned readmission
Source: PLoS One. 2022 Jul 15;17(7):e0271331. doi: 10.1371/journal.pone.0271331 (PMC9286269; doi:10.1371/journal.pone.0271331)
Supplement: S2 Appendix — Detail of the main steps that compose the procedures mentioned in the methodology section. (PDF) [file pone.0271331.s002.pdf]

Decision support through risk cost estimation in 30-day hospital unplanned readmission.

S2 Appendix: Additional information on the used methods

Laura Arnal[1◑*], Pedro Pons-Suñer[1◑], J.Ramón Navarro-Cerdán[1◑], Pablo Ruiz-Valls[1◑], Mª Jose Caballero Mateos[2◑], Bernardo Valdivieso Martínez[2◑], Juan-Carlos Perez-Cortes[1◑],

1 Instituto Tecnológico de Informática (ITI), Universitat Politècnica de València, Camino de Vera, s/n, 46022 València, Spain
2 Health Research Institute of La Fe University Hospital, Fernando Abril Martorell, Torre A, s/n, 46026 València, Spain

◑These authors contributed equally to this work.
* larnal@iti.es

Methodology summary

In this additional section we provide a more detailed description of the main steps mentioned in the methodology section. In the form of two pseudocode algorithms, we present:

- Algorithm 1: a general data preprocessing pipeline which involves filtering functions based on various different criteria and finalising with a standarization step.
- Algorithm 2: the main algorithm encompassing training and testing a machine learning model followed by calibration and scaling steps. Predicted risks and true labels are compared at the end to get performance metrics.

Algorithm 1 Dataset general preprocessing pipeline

```
imputer_models ← (constant_imputer(fill="unknown"),
        constant_imputer(strategy="most_frequent"),
        constant_imputer(fill=0),
        iterative_imputer(estimator=BayesianRidge))
vars_per_imputer ← (categorical_features,
        binary_features,
        tratments_features, consumptions_features, comorbidity_features,
        laboratory_features)
nan_threshold ← 0.3
imputation_opts ← imputer_models, vars_per_imputer, nan_threshold

var_threshold ← 0.99
corr_threshold ← 0.9

procedure PREPROC_PIPELINE(data, imputation_opts, nan_threshold, var_threshold, corr_threshold)
    ▷ Remove columns with % missing values above threshold
    data ← filter_missing_values(data, nan_threshold)

    ▷ Remove laboratory columns with % missing values above threshold/2 and imputation score below 0.5
    for col in laboratory_columns do
        ▷ Replace a portion with NaN and impute. Then get R2 between true and imputed
        col_score ← r2_imputation_score(data, col)
        nan_percentage ← sum(data[col]==NaN) / len(data.rows)
        if col_score < 0.5 and nan_percentage > nan_threshold/2 then
            data ← data.drop_column(col)
        end if
    end for

    ▷ Impute missing values in remaining columns
    models, vars_per_model, threshold ← imputation_opts
    for model, vars in zip(models, vars_per_model) do
        vars ← vars ∩ data.columns
        data[vars] ← model.transform(data[vars])
    end for

    ▷ One-Hot-Encoding of categorical features
    cat_features ← get_categorical_features(data)
    data ← ohe_transform(data, cat_features)

    ▷ Remove invariant columns
    data ←remove_constant_columns(data, var_threshold)

    ▷ Remove columns for pairs with high correlation
    data ←remove_correlated_columns(data, corr_threshold)

    ▷ Standardize columns
    data ←standard_scaling(data)

    return data
end procedure
```

Algorithm 2 Main procedure for model training and testing

```
procedure MAIN_PROCEDURE(data, preproc_opts, base_model, calibrator_model, scaling_regressor)
    ▷ Preprocess data
    X, y ← split_data_target(data)
    imputation_opts, var_threshold, corr_threshold ← preproc_opts
    X ← preproc_pipeline(X, imputation_opts, var_threshold, corr_threshold)

    y_true_all ← [ ]                    ▷ Placeholder to concatenate all true labels
    y_pred_all ← [ ]                    ▷ Placeholder to concatenate all predict data

    ▷ Split data in 10 major train-test folds
    k_fold ← KFold(n_splits=10)
    for traincalib_idx, test_idx in kfold.split(X, y) do
        x_traincalib, y_traincalib ← X[traincalib_idx], y[traincalib_idx]
        x_test, y_test ← X[test_idx], y[test_idx]

        ▷ Further split traincalib in 10-fold train and calib sets
        calib_train_data ← [ ]
        calib_true_data ← [ ]
        for train_idx, calib_idx in kfold.split(x_traincalib, y_traincalib) do
            x_train, y_train ← x_traincalib[train_idx], y_traincalib[train_idx]
            x_calib, y_calib ← x_traincalib[calib_idx], y_traincalib[calib_idx]
            base_model.fit(x_train, y_train)
            calib_train_data ← calib_train_data ∪ base_model.predict_proba(x_calib)
            calib_true_data ← calib_true_data ∪ y_calib
        end for
        ▷ Fit calibrator and scaler with all calib data
        calibrator.fit(calib_train_data, calib_true_data)
        train_calibrated_probas ← calibrator.predict(calib_train_data)
        train_percentiles ← to_percentiles(train_calibrated_probas)
        scaling_regressor.fit(train_calibrated_probas, train_percentiles)

        ▷ Fit base model with all traincalib data and transform to percentile range (0-100)
        base_model.fit(x_traincalib, y_traincalib)
        y_base_pred ← base_model.predict_proba(x_test)
        y_calib_pred ← calibrator.predict(y_base_pred)
        y_prctl_pred ← scaling_regressor.predict(y_calib_pred)

        ▷ Add fold true labels and predicted percentiles to major arrays
        y_true_all ← y_true_all ∪ y_test
        y_pred_all ← y_pred_all ∪ y_prctl_pred
    end for
    ▷ Get desired metrics and return
    return get_metrics(y_true_all, y_pred_all)
end procedure
```
